# Congenital Extra-Ventricular (Ganglio)Neurocytoma of the Brain Stem: A Case Report

**DOI:** 10.3389/fped.2018.00108

**Published:** 2018-05-11

**Authors:** Marta Piras, Evelina Miele, Angela Di Giannatale, Giovanna S. Colafati, Francesca Diomedi-Camassei, Maria Vinci, Emmanuel de Billy, Angela Mastronuzzi, Andrea Carai

**Affiliations:** ^1^Department of Hematology, Oncology and Stem Cell Transplantation, Bambino Gesù Children's Hospital (IRCCS), Rome, Italy; ^2^Neuroradiology Unit, Department of Imaging, Bambino Gesù Children's Hospital (IRCCS), Rome, Italy; ^3^Department of Pathology, Bambino Gesù Children's Hospital (IRCCS), Rome, Italy; ^4^Neuro-oncology Unit, Department of Hematology, Oncology and Stem Cell Transplantation, Bambino Gesù Children's Hospital (IRCCS), Rome, Italy; ^5^Neurosurgery Unit, Department of Neuroscience and Neurorehabilitation, Bambino Gesù Children's Hospital (IRCCS), Rome, Italy

**Keywords:** brain tumor, (Ganglio)neurocytoma, extraventricular neurocytoma, pediatric, brainstem

## Abstract

Extraventricular neurocytoma (EVN) is an extremely rare tumor of neuroglial origin with a tendency toward ganglionic or glial differentiation. In the 2016 World Health Organization Classification, EVN was classified as a grade II tumor and described as a neoplasm with good outcome. However, the presence of cellular atypia is an important unfavorable prognostic factor. Here, we describe the first case of a patient with a congenital EVN localized in the brainstem. After a sub-total resection, his disease rapidly progressed despite several chemotherapies, including molecular targeting approaches. He died 13 months after diagnosis. In conclusion, we report an atypical case of EVN presenting an extremely aggressive behavior, despite the absence of cellular atypia. The brainstem origin and the age of the patient may have represented two important prognostic factors for our patient.

## Introduction

Brain tumors are extremely rare in the first year of life, accounting for less than 2% of brain neoplasms at all pediatric ages [1]. The definition of “congenital brain tumor” (CBT) is subject to debate, with several classifications described in the literature [2]. However, the 2 main criteria taken into account for the diagnosis of a CBT are the age of the child at the onset of symptoms (first 3 months of age) [3] and the histology of the tumor which differs from the one observed for older patients [2].

Neurocytomas are rare brain tumors associated with neuronal differentiation, which develop mainly in young adults aged 25–35 years old and rarely in children with no case, to our knowledge, being reported as congenital [4]. Neurocytomas are located within the ventricular system (CN, “*central neurocytomas”*) or, less commonly, within the extra-ventricular region (EVN, “*extra-ventricular neurocytoma”)* of the central nervous system. EVN differs from CN by having a more heterogeneous and complex histological appearance. When compared to CN, EVN is less cellularized, and shows a pronounced tendency toward glial or ganglionic differentiation in part of the tumor, hence providing the name of extra-ventricular (ganglio)neurocytoma [5].

EVNs are defined as grade II tumors according to the WHO classification [6]. However, EVNs can be divided into typical and atypical sub-groups, depending on their histological features and aggressiveness. The typical EVNs represent the less-aggressive variants of the disease. In contrast, the atypical EVNs are aggressive tumors characterized by an elevated mitotic and proliferative index and/or high vascularization and necrosis [7]. Another factor indicative of unfavorable prognosis for EVNs is cellular atypia [8], although there are reports on EVNs with atypia characteristics for patients who have excellent disease control, and, on the other hand, EVNs without sign of atypia, for patients who present a poor overall outcome [9].

We report the first case of congenital EVN localized in the brainstem of a 3-month-old patient. The patient presented a dismal outcome despite multimodal therapy and absence of cellular atypia.

## Case description

### History

A 3-month-old male was referred to our hospital for the management of a congenital intracranial mass and infective endocarditis in the bicuspid aortic valve.

**Figure 1:**
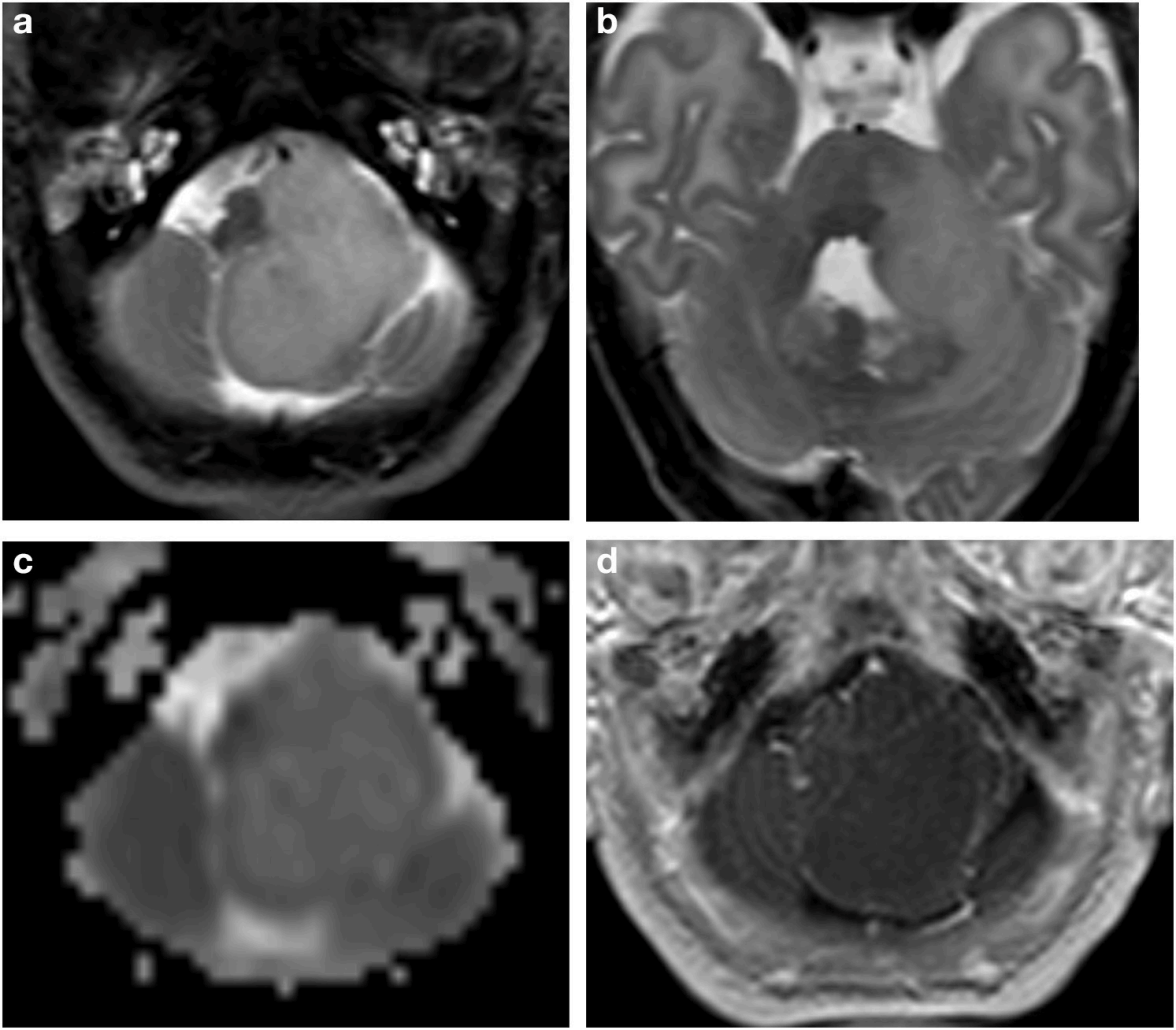
Brain MRI. Axial T2w **(a,b)** ADC map **(c)** and Gd T1w **(d)** images. Extensive hyperintense mass in the left cerebellopontine angle extended upward into the cerebellum with lower peripheral ADC values and subtle peripheral linear irregular enhancement after gadolinium injection. The fourth ventricle, medulla oblongata and the cerebellum are dislocated and compressed.

At birth, the child presented lower limb weakness, left seventh nerve palsy and left neck swelling, due to his cardiac problem, associated with a head turning difficulty. A magnetic resonance imaging of the brain showed a left pontine-bulbar lesion extended upward into the cerebellum. The fourth ventricle and the cerebellar vermis was dislocated (Figure 1). A few days after the diagnosis, he presented fever associated to *Staphylococcus aureus* bacteremia. An echocardiogram showed a vegetative endocarditis in the bicuspid aortic valve with an ejection fraction of 45%. The patient received antibiotic therapy for this infection.

After transfer to our center, he underwent aortic valve replacement via Ross Procedure with no complications. He presented a neurological deterioration with a left sided hemiparesis, dysphagia and recurrent episodes of apnea.

One month after the cardiac surgery, he underwent suboccipital craniotomy in the prone position with intraoperative neuronavigation and neurophysiologic monitoring. The surgery was interrupted during resection of the infiltrating bulbar component because of sustained bradycardia and arterial hypertension and only a sub-total resection (STR) was performed. He presented a stable neurological status after surgery but received a tracheostomy for a mild pulmonary insufficiency.

### Pathological findings

**Figure 2:**
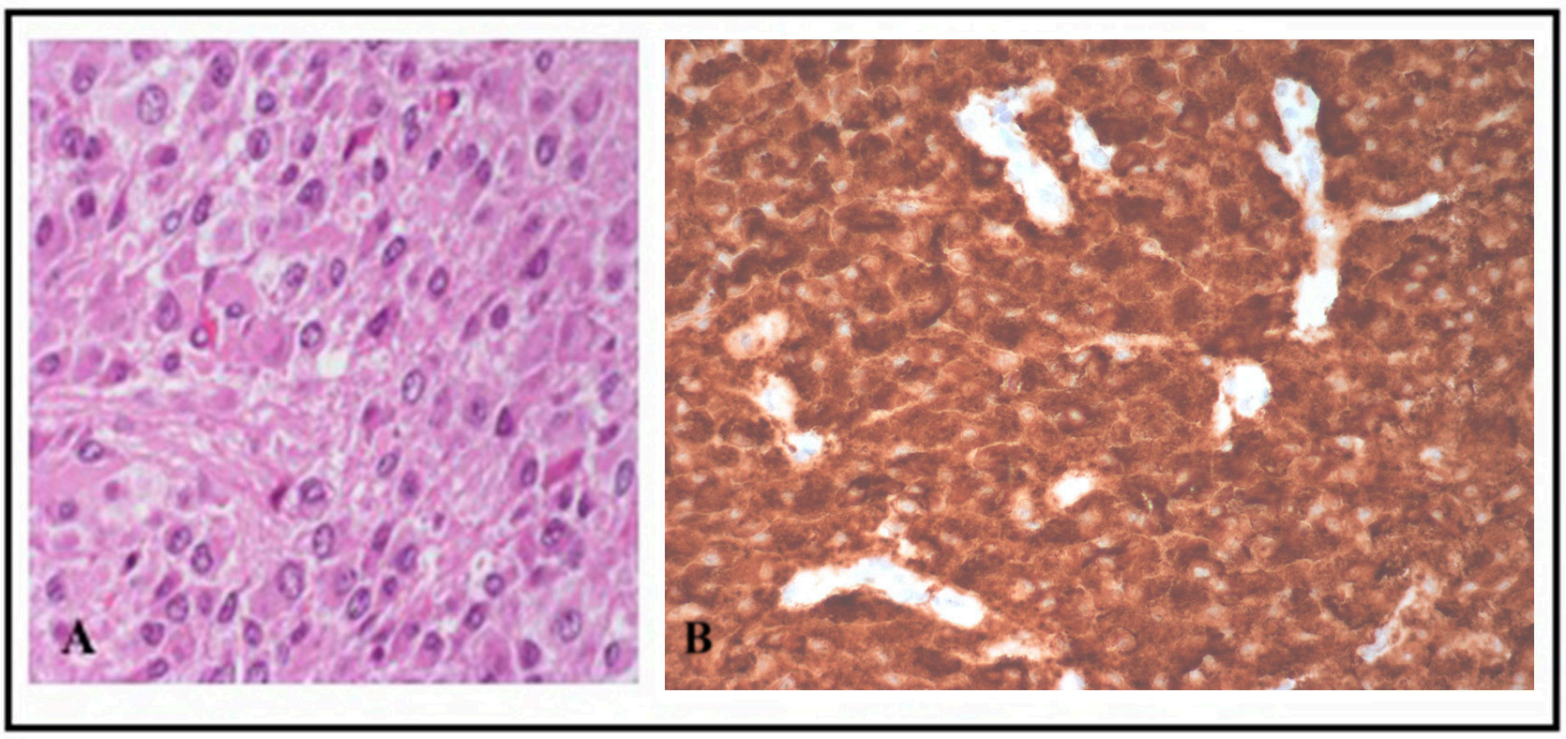
**(A)** Hematoxylin&Eosin staining shows a diffused, monomorphic, round cell proliferation with various degrees of ganglioid differentiation. **(B)** Strong positivity for synaptophysin immunostain.

Histology showed proliferation of small/medium-size round cells with a central round nucleus, finely speckled chromatin, and small nucleolus. Ganglioid cells, intermediates between neurocytes and ganglion cells, were evenly distributed and differentiation toward fully mature ganglion cells was observed. Neoplastic cells were strongly positive for synaptophysin and neurofilaments, mildly positive for Neu-N, and negative for GFAP and CD34, indicating a neuronal nature of the tumor. Additional immunohistochemistry revealed weak positivity for p53 (<5%), mTor, phospho-mTor and EGFR. Ki67 was low (1% with some areas up to 3–4%). Mitoses were 3–4/10 HPF. BRAF^v600E^ mutation was assessed by DNA sequencing and resulted positive. A diagnosis of extra-ventricular (ganglio)neurocytoma was then formulated and the tumor was classified as grade II according to WHO 2016, with a particular mention of the focal higher proliferation index (Figure 2).

### Postoperative course

Considering the young age of the patient and the sub-total resection, we decided to perform an adjuvant therapy. After two cycles with high-dose carboplatin and etoposide, Magnetic Resonance Imaging (MRI) showed a local disease progression. A treatment with the mTOR inhibitor, Everolimus, as single agent was started but no tumor response was observed. Despite further treatments with irinotecan, temozolomide and then the BRAF^V600E^ inhibitor Vemurafenib, the MRI showed a gradual increase of tumor size associated with hydrocephalus. The patient underwent a ventriculo-peritoneal shunt. The number and duration of desaturation episodes increased due to the involvement of the medullary respiratory center. Intermittent mechanical ventilation and total parenteral nutrition were necessary.

The child died of a cardiorespiratory arrest thirteen months after diagnosis.

## Discussion

EVN is included in the 2016 “*WHO classification of tumors of the central nervous system*” in the “*Neuronal and mixed neuronal-glial tumors”* group and classified as grade II [6]. Most of EVNs are supratentorial with few cases reported as cerebellar or spinal, and only 3 described in the pons [10]. EVN incidence is unknown but it is certainly a rare disease with just over 100 cases found in the literature. EVN arises predominantly in young adults (median age of 25 years old) [11], and there are only 43 cases of pediatric EVNs reported so far and summarized in Table 1.

**Table 1:** Previously reported extra-ventricular neurocytoma in children.

**Authors**	**Age (years)**	**Tumor location**	**Surgery**	**Atypical/Typical**	**Status**
Agarwal et al. [15]	16	Spinal cord	STR	Atypical	Alive
Ahmad et al. [17]	15	Brainstem, cerebellum	Biopsy	Typical	Alive
Brat et al. [18]	5–18 (7 patients)	Cerebrum	STR/GTR	n.d	Alive/ nd
Buchbinder et al. [19]	1	Frontal	GTR	Atypical	Dissemination
Choi et al. [7]	8	Frontal lobe	STR + second surgery	n.d	Alive
Garber and Brockmeyer [20]	8	Frontal	GTR	n.d	Alive
Ghosal et al. [21]	9	Fronto-parietal	n.d	Atypical	n.d
Giangaspero et al. [22]	5–18 (4 patients)	Frontal, occipital, temporal, parietal, hypothalamus	1 STR/ 3GTR	Typical	1 died (STR)/ 3 alive
Hamilton [23]	11	Frontal	n.d	n.d	n.d
Han et al. [14]	2–4 (3 patients)	Frontal, fourth ventricul	GTR	Atypical	Succumbed, alive
Kawano [24]	3	Frontal lobe	STR	Atypical	Alive
Makhdoomi et al. [25]	5	Cerebellum	GTR	Typical	Alive
Messina et al. [26]	10 (2 patients)	Frontal lobe	GTR	Typical; atypical	Alive
Mpairamidis et al. [27]	3	Parietotemporal	GTR	Atypical	Alive
Myung et al. [28]	9	Frontal	STR	Atypical	Recurrence
Nishio et al. [29]	1–2 (2 patients)	Frontal-temporal	STR	n.d	Alive
Polli et al. [30]	6–15 (2 patients)	Spinal cord	GTR	n.d	Alive/ Died
Psarros et al. [31]	1	Spinal cord	Biopsy	Typical	n.d
Raja et al. [32]	7	Occipital	GTR	Atypical	Dissemination
Singh et al. [33]	8	Spinal cord	STR	Atypical	Alive
Shuster et al. [34]	8 months	Talamic-diencephalic	n.d	Atypical	n.d
Tortori-Donati et al. [5]	9	Temporal	GTR	Typical	Alive
Xiong et al. [35]	13	Parietal-occipital	n.d	Atypical	alive
Yang et al. [36]	2	Frontal	n.d	n.d	n.d
Yi et al. [37]	10–13 (4 patients)	Cerebral hemisphere, cerebellum and thalamus	n.d	Typical	n.d
Zacharoulis et al. [9]	1–14 (5 patients)	n.d	GTR (3 patients); STR (2 patients)	Atypical (3 patients); typical (2 patients)	Alive

*GTR, gross total resection; STR, sub-total resection; n.d, not defined; TPN, total parental nutrition*.

To our knowledge, we describe here the first case of a congenital EVN. Therefore, EVN could be added to the list of other known CBTs, including teratomas, choroid plexus papillomas, embryonal tumors, infantile desmoplastic astrocytomas/gangliogliomas and craniopharyngiomas [2]. The EVN described here presented characteristics of (ganglio)neurocytomas suggested by the presence of ganglioid intermediates and fully differentiated ganglion cells. The disease did not show a detectable sign of cellular atypia, a factor normally considered as an unfavorable prognosis for EVN [9]. However, we defined it as atypical because of the high proliferation index (Ki67>2%) observed in some tumor areas, a feature associated with a high recurrence rate [12]. Our case also presented a mutation in the BRAF gene never described before for EVN and rarely detected in pediatric brain tumors [13].

Because of the rarity of the EVNs, few data are available regarding the therapeutic strategies used to treat patients [14]. The use of radiotherapy in children is subject to debate because of the poor survival benefit [15] and the undesirable effects observed in the developing brain, such as stroke and neurocognitive deficits [16]. For this patient the radiotherapy option was not considered because of the young age and the presence of recurrent episodes of apnea. Gross total resection (GTR), which represents the mainstay of treatment for EVN [14], was not possible because of the brainstem localization of the tumor, so only an STR was performed. The main chemotherapeutic regimens used to date for EVNs treatment include cisplatin/etoposide/cyclophosphamide, procarbazine/CCNU/vincristine and ifosfamide/carboplatin/etoposide [9]. In our case, following an STR, two cycles of high dose of carboplatin and cisplatin, and the use of targeted therapies including topoisomerase, mTOR and BRAF^V600E^ inhibitors, the disease progressed and the young patient died 13 months after diagnosis.

## Conclusions

To our knowledge, this is the first report of a congenital EVN. The disease, localized in the brainstem, presented an aggressive behavior and progressed rapidly, leading to death despite the absence of unfavorable histology with the exception of a focal high proliferation index. The impossibility to achieve a GTR and the critical localization of the tumor are two potentially significant factors for the prognosis of patients who develop an EVN.

## Ethics statement

This study was carried out in accordance with the recommendations of the Internal Review Board of the Bambino Gesù Ospedale Pediatrico with written informed consent from all subjects. All subjects gave written informed consent in accordance with the Declaration of Helsinki. The protocol was approved by the Internal Review Board of the Bambino Gesù Ospedale Pediatrico.

## Informed consent

The authors declare that written informed consent was obtained from the patient's parents for publication of this case report.

## Authors contributions

MP: design of the work, structuration, interpretation of the data, writing; AM: conception (ideation), structuration, acquisition of the data, structuration, revision, final approval to be published; EM: acquisition of the data, structuration, revision; EdB and AD: revision; MV: acquisition of the data, elaboration of the data; GC: acquisition of the data, acquisition and elaboration of the images; FD-C: pathological findings; AC: conception, structuration, acquisition of the data, revision.

### Conflict of interest statement

The authors declare that the research was conducted in the absence of any commercial or financial relationships that could be construed as a potential conflict of interest.
